# SALL4 suppresses PTEN expression to promote glioma cell proliferation via PI3K/AKT signaling pathway

**DOI:** 10.1007/s11060-017-2589-3

**Published:** 2017-09-08

**Authors:** Chuanjin Liu, Haibin Wu, Yanyan Li, Liang Shen, Renchun Yu, Hongwei Yin, Ting Sun, Chunming Sun, Youxin Zhou, Ziwei Du

**Affiliations:** Neurosurgery & Brain and Nerve Research Laboratory, The First Affiliated Hospital of Soochow University, 188 Shizi Street, Suzhou, 215006 Jiangsu People’s Republic of China

**Keywords:** Glioma, SALL4, PTEN, PI3K/AKT, Proliferation

## Abstract

Spalt-like transcription factor 4 (SALL4), a oncogene, is known to participate in multiple carcinomas, and is up-regulated in glioma. However, its actual role and underlying mechanisms in the development of glioma remain unclear. The present study explored the molecular functions of SALL4 in promoting cell proliferation in glioma. The expression level of SALL4 in 69 human glioma samples and six non-tumor brain tissues was determined using real-time polymerase chain reaction (PCR). Then, we transfected U87 and U251 cell lines with siRNA, and assessed cellular proliferation and cell cycle to understand the function of SALL4, and the relationship between SALL4, PTEN and PI3K/AKT pathway. PCR confirmed that the expression of SALL4 was higher in the glioma samples than non-tumor brain tissues. Cellular growth and proliferation were dramatically reduced following inhibition of SALL4 expression. Western blot showed increase in PTEN expression when SALL4 was silenced, which in turn depressed the activation of PI3K/AKT pathway, suggesting that PTEN was a downstream target of SALL4 in glioma development. Therefore, SALL4 could act as a proto-oncogene by regulating the PTEN/PI3K/AKT signaling pathway, thereby facilitating proliferation of glioma cells.

## Introduction

Malignant glioma has the highest incidence among human primary brain tumors, and is characterized by high mortality rate, recurrence and malignancy. In spite of comprehensive therapies, the prognosis and survival of glioma patients remain poor [1]. Malignant growth, high proliferation of glioma cells and high infiltration that makes full surgical resection impossible are the predominant reasons for poor prognosis and survival. Like other types of tumors, the causes of glioma are varied, and include activation of oncogenes. The embryonic stem cell (ESC) gene SALL4 has been recently identified as a new target for cancer therapy.

SALL4 is the human homolog of Drosophila spalt (sal) mapped to chromosome 20q13 and encodes a C2H2 zinc-finger transcription factor [2], which is important for maintenance of pluripotent and self-renewal properties of ESCs [3]. With the same function of oncogenes, SALL4 participates in cell proliferation, apoptosis, cycle, invasion, drug resistance, and the formation and evolution [4–7] of multiple human solid tumors, such as hematopoiesis, hepatocellular carcinoma, lung cancer, myelodysplastic syndrome [8–10].

Phosphatase and tension homolog (PTEN), is a tumor suppressor whose expression is very low in various human tumors [11–13]. The PI3K/AKT signaling pathway is a well-known pathway in the regulation of tumorigenesis, and is significantly activated in glioma [14]. PTEN contributes in antagonizing PI3K [15], thereby weakening AKT activation [16], which could suppress down-stream products thereby inducing cell cycle arrest in the G1 phase by increasing ki-67 expression [15] and decreasing cyclin D1 expression [17].

Based on the important function of PI3K/AKT signaling in glioma development [18, 19] and the crosstalk between SALL4 and PTEN [20], we found that SALL4 mRNA expression was significantly higher in glioma specimens than in non‑cancerous brain samples. SALL4 expression may promote the formation of glioma, but the underlying mechanism remains unclear. The present study was based on the hypothesis that SALL4 could suppress PTEN, thereby strengthening PI3K/AKT signaling.

## Materials and methods

### Human tissue samples

Specimens were collected from patients who underwent surgical removal of brain tumors at the Department of Neurosurgery, Brain and Nerve Research Laboratory of The First Affiliated Hospital of Soochow University (Suzhou, China) from 2009 to 2012. Six non-tumor brain samples were collected from patients without brain tumors who underwent traumatic brain injury or arteriovenous malformation, which needed resection of a small part of their brain tissues to lower the intracranial hypertension and increase treatment outcome. Thirty-seven female and 32 male glioma patients were included. Among them, 17 had grade II (diffuse astrocytoma), 26 had grade III (anaplastic astrocytoma), and 26 had grade IV (primary brain glioblastoma), according to the 2007 WHO classification system. The mean age of the patients at the time of surgical resection were 46.9 years for men and 44.9 years for women. The mean age was 40.62 ± 15.64 years for grade II, 43.89 ± 15.21 for grade III and 48.12 ± 14.97 years for grade IV. All samples were collected and immediately stored in liquid nitrogen after resection. This study was approved by the local ethics committee of The First Affiliated Hospital of Soochow University, and all patients gave informed consent for the usage of their samples in the study.

### Cell cultures and treatments

The U87MG and U251MG were obtained from the Cell Bank Type Culture Collection of the Chinese Academy of Sciences (Shanghai, China). Cells were maintained in DMEM (Hyclone, Thermo Fisher Scientific, USA) supplemented with 10% FBS (Gibco, Invitrogen, USA) at 37 °C under a humidified atmosphere of 5% CO_2_.

### siRNA transfection

For down-regulation of SALL4, 50 pmol/l SALL4-siRNA, filtrating the best one from three different kings of SALL4-siRNA (siRNA-1:5-CCGAAAGCAUCAA GUCAAATT-3;5-UUUGACUUGAUGCUUUCGGTT-3. siRNA-2:5-GUCUCUGGAUGCCUGAAATT-3; 5-UUUCAAGGCAUCCAGAGACTT-3. siRNA-3:5-GUGGCCAACACUAAUGUGATT-3; 5-UCACAUUAGUGUUGGCCACTT-3) were transfected into the cells using Lipofectamine 2000 (invitrogen) according to the manufacturer’s instructions. The siRNA vectors were are purchased from Shanghai Genepharma Co., Ltd. The transfection rates of two human glioma cell lines U87 and U251 were determined by flow cytometry. Transfection ratio >80% was used for the experiments (the U87 transfection efficiency was 97.8% and the U251 transfection efficiency was 99.8%).

### Quantitative RT-PCR

RNA from cells and specimens was extracted by TRIzol reagent (Invitrogen, USA), and quantified by spectrophotometer. Only mRNA with 260/280 ratios of 1.9–2.0 were used for the experiments. Relative levels of mRNA were examined using SYBR green real-time quantitative RT-PCR (qRT-PCR) (LightCycle r480 Roche, Switzerland), and normalized by GAPDH mRNA. qRT-PCR results were calculated using the 2^−△△CT^ method, data analyses were performed in triplicate. Three times independent experiments are repeated and all data are presented as means and standard errors of the means.

### Flow cytometric analysis

Glioma cell lines U87 and U251 were cultured in 6-well plates, at a cell density of 2 × 10^5^ per well, to ensure a transfection density of 80–90%. After culturing for 24 h, transfection efficiency was detected. Cells were transfected with siRNA negative control, SALL4-siRNA and SALL4-siRNA-bpv (PTEN inhibitor bpv were add into SALL4-siRNA group), and incubated for 48 h before the cell cycle test. Both transfection efficiency and cell cycle were measured by flow cytometer and repeated three times this experiments.

### CCK-8 assay

The proliferation of cells was detected by cell counting kit (CCK-8, Dojindo, China). siRNA negative control, SALL4-siRNA and SALL4-siRNA-bpv were transfected into the U87 and U251 glioma cells. After incubating for 12, 24, 48 and 72 h, CCK-8 was added and the absorbance was measured at 450 nm (Thermo, USA) after incubating for another 2 h. The OD was calculated as mean ± SD of three measurements per sample.

### Western blotting

Fourty-eight hours after SALL4-siRNA and negative control siRNA transfection or 24 h before addition of PTEN inhibitors pten (bpv), the whole protein lysate was prepared from cells by radioimmunoprecipitation assay lysis buffer containing 50 mM Tris/HCl pH 7.5, 0.1% sodium dodecyl sulfate (SDS), 1% Triton-X 100, 0.5–1% sodium deoxycholate, 150 mM sodium chloride, and protease inhibitors (Roche). Bicinchoninic acid (BCA) protein assay kit (Beyotime Institute of Biotechnology) was used to calculate the protein concentration. Equal amounts of protein samples were separated with 6–10% SDS-PAGE and transferred onto nitrocellulose membrane, under constant voltage of 60 V. The membrane was blocked with 5% bovine serum albumin (BSA) (Amresco, USA) for 2 h at room temperature, and then incubated with 1:1000 primary antibodies overnight at 4 °C and 1:3000 HRP-conjugated secondary antibody (Beyotime Institute of Biotechnology) for 2 h followed by washing with PBST three times for 5 min each time. The signal was detected using enhanced chemiluminescence (ECL) (Thermo). The primary antibodies used in this study were anti-SALL4, anti-cyclin D1 (Abcam, Japan), anti-PTEN, anti-PI3K, anti-p-PI3K, anti-AKT, anti-p-AKT, and anti-GAPDH (CST, USA). Repeated three times this experiments.

### Immunofluorescence analysis

U87 and U251 cells were transfected with SALL4-siRNA, negative control and SALL4-siRNA-bpv, treated with bpv, fixed with 4% paraformaldehyde for 30 min, and blocked with BSA (Amresco) for 30 min. The cells were incubated with anti-PTEN, anti-p-PI3K (CST, USA) primary antibodies at 4 °C overnight, and then incubated with tetramethylrhodamine isothiocyanate-labeled secondary antibody (diluted 1:500) at 37 °C for 30 min. The cells were stained with DAPI, and imaged with a fluorescence microscope (OLYMPUS BX50/BXFLA/DP70; Olympus Co., Japan). Three times independent experiments are repeated.

### Statistical analysis

Statistical analyses were conducted with GraphPad, PRISM4.0 software (GraphPad, USA) and SPSS 13.0 software (SPSS Inc., USA). Student’s *t*-test or ANOVA were used to test the difference between the groups. *P* < 0.05 was considered to be statistically significant.

## Results

### Expression of SALL4 mRNA in glioma samples and non-tumor brain tissues

**Fig. 1:**
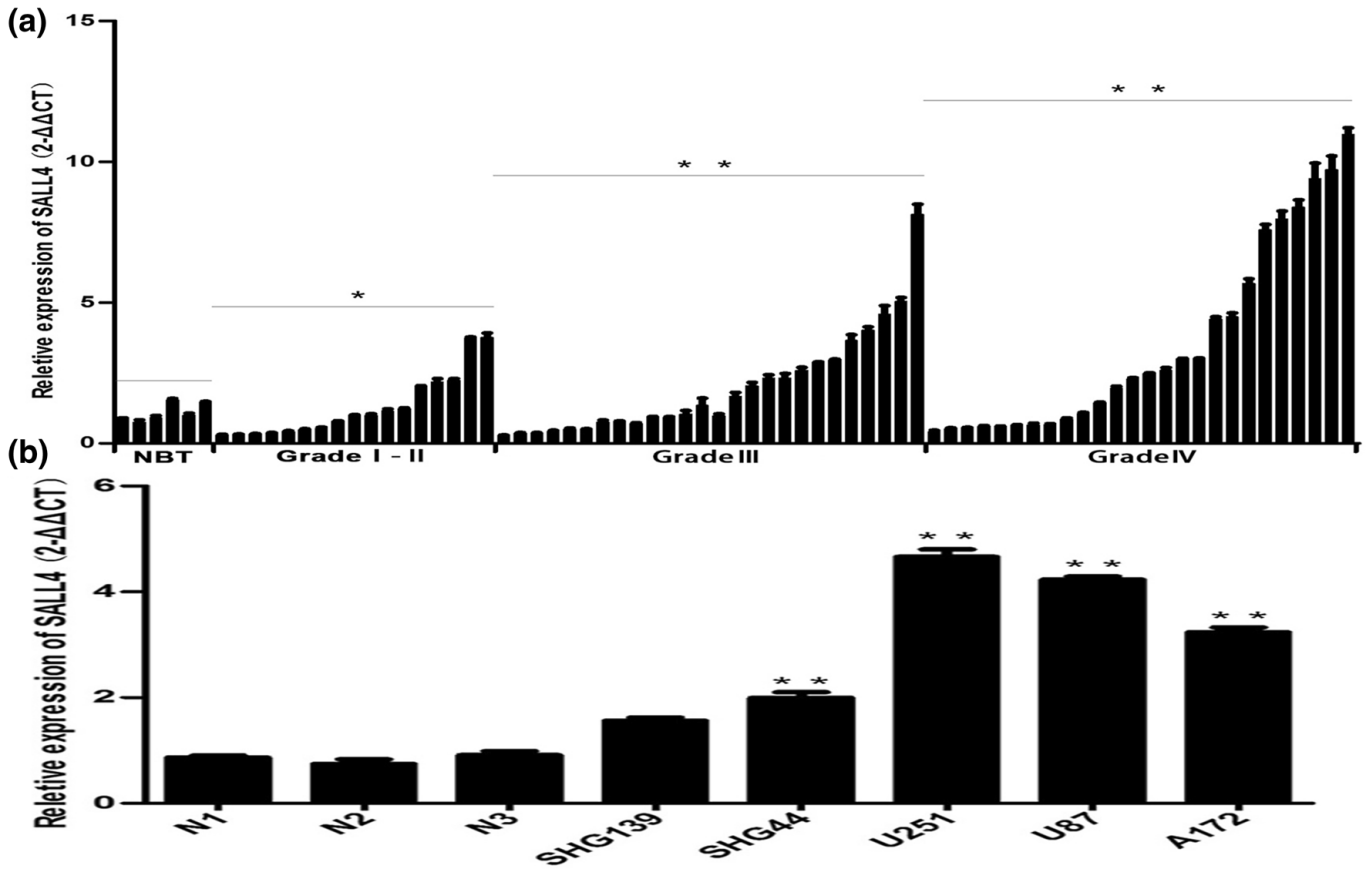
**a** shows qRT-PCR analysis (data were reported as 2^−△△CT^) of the expression of SALL4 in six non-tumor brain tissues and 69 glioma tissues. SALL4 expression was markedly higher in glioma than in non-tumor brain tissues and increased with the increase in degree of malignancy in glioma. **b** SALL4 expression in glioma cell lines was up-regulated as compared to normal brain tissues (NBT including N1, N2 and N3). N1, N2 and N3 small nuclear RNA was used as an internal control. *P < 0.05, **P < 0.01. Higher grade glioma cells had higher level of SALL4

qPCR was performed to quantify the expression of SALL4 in 69 glioma samples and six non-tumor brain tissues. The expression of SALL4 was higher in the glioma samples than in the non-tumor brain tissues, and increased with the increase in degree of malignancy in glioma (*P* < 0.05; Fig. 1a). SALL4 level was detected in different types of glioma cell lines(SHG139, SHG44, U87, U251, A172) and the more malignant the cell, the higher was the expression of SALL4. U87 and U251 cell lines were selected for the present study (Fig. 1b).

### Relationship between SALL4 and PTEN in glioma

**Fig. 2:**
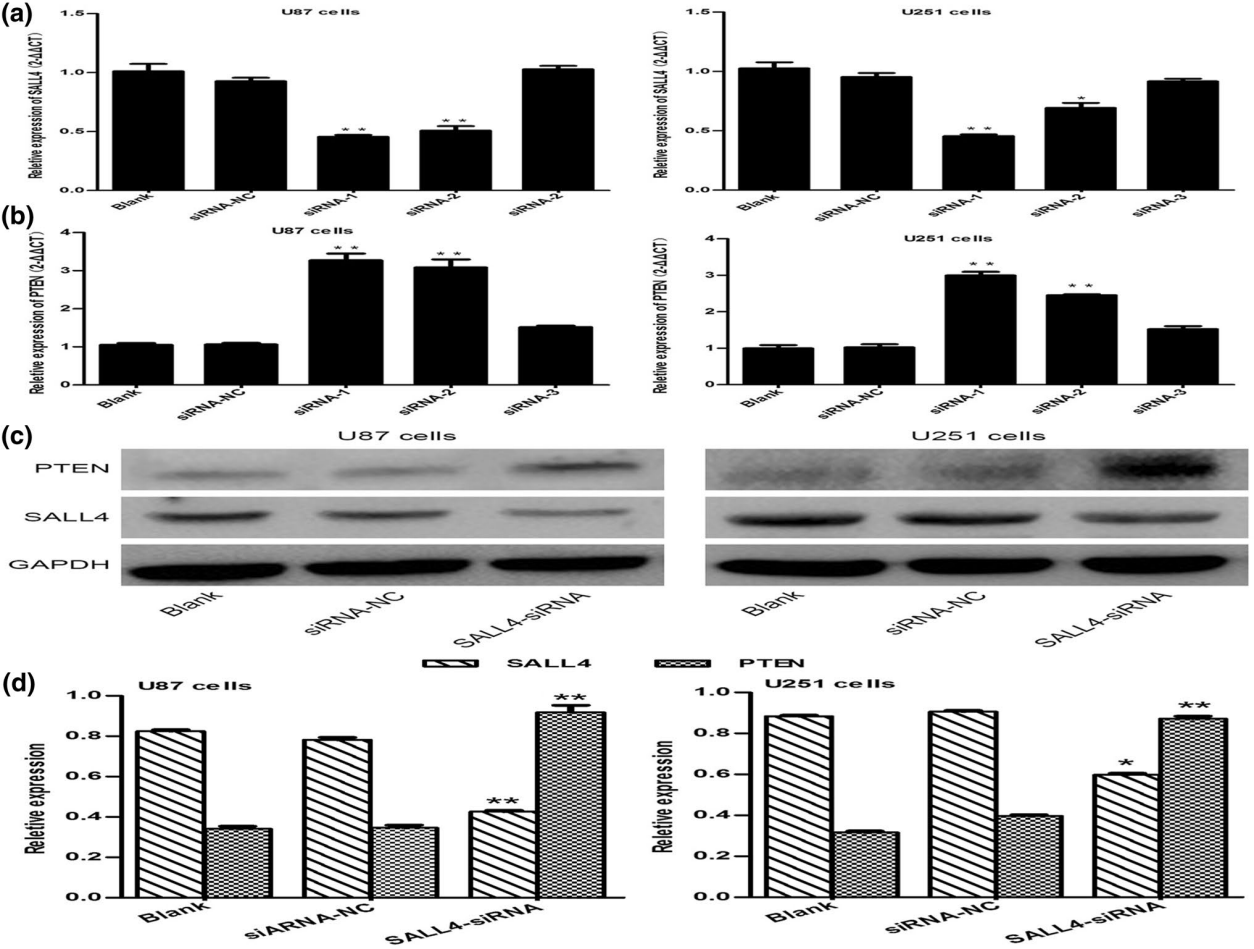
**a** The inhibitory effect of three siRNA sequences on SALL4 level in U87 and U251 cells. **b** Blocking SALL4 with different siRNA sequences upregulated PTEN in siRNA level. **c, d** Western blot showed that suppression of SALL4 by SALL4-siRNA increased PTEN protein expression (*P < 0.05)

PTEN was shown to be regulated by SALL4 in some types of tumors. Li found the cancer suppressor gene PTEN was obviously down-regulated in glioma [21]. To estimate the effect of SALL4 on PTEN, firstly, the most efficient siRNA was selected from the three siRNA sequences (*P* < 0.05; Fig. 2a). Secondly, mRNA of U87 and U251 cells transfected with SALL4-siRNA were used for qRT-PCR, which showed that the expression of PTEN was apparently up-regulated when SALL4 was blocked (*P* < 0.05; Fig. 2b), implying a negative correlation between PTEN and SALL4. Western blot also demonstrated that PTEN protein was significantly higher in cells transfected with SALL4-siRNA as compared to siRNA negative control and blank groups (*P* < 0.05; Fig. 2c, d). Based on these findings, the expression of SALL4 might suppress the mRNA and protein levels of PTEN.

### siRNA-SALL4 reduces proliferation of glioma cells

**Fig. 3:**
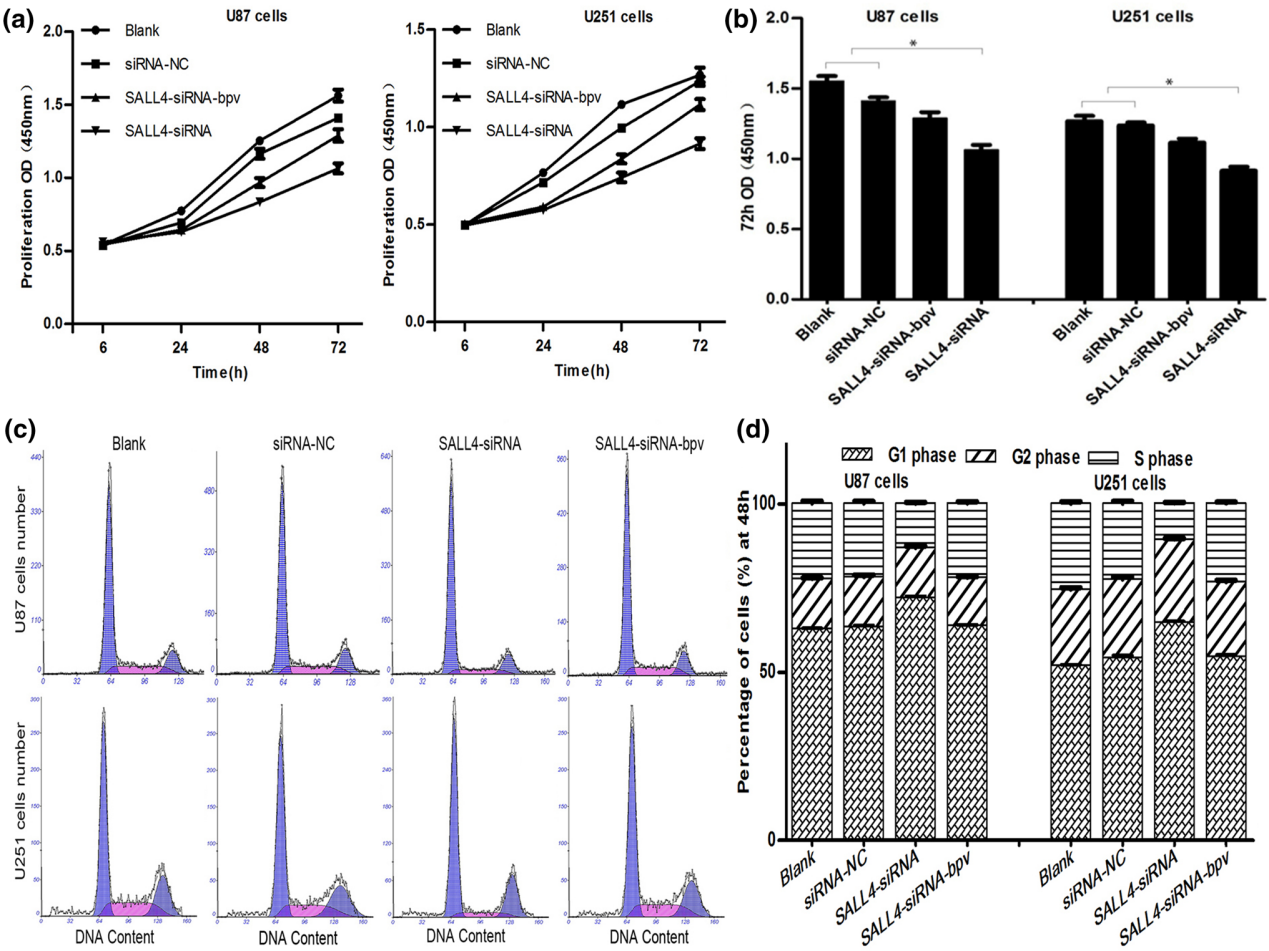
**a** Cell counting kit (CCK-8) was used to detect cellular proliferation in U87 and U251 cells at 6, 24, 48, and 72 h after siRNA transfection. **b** Shows the OD value at the time of 72 h and data were reported as means ± SD (*P < 0.01). **c, d** Blocking SALL4 induced U87 and U251 at the time of 48 h, cell cycle arrest in the G1 phase, which decreased cells in the S phase, increased cells in the G1 phase. Cell cycle in the cells treated with SALL4-siRNA and phen (bpv) were similar to blank and negative control groups (*P* < 0.05)

Rapid proliferation of glioma cells leads to poor prognosis and short survival. Cellular proliferation assays in U87 and U251 cells were conducted using CCK-8 to explore the influence SALL4 on growth of glioma cells. SALL4 expression was decreased after transfection with SALL4-siRNA, which in turn resulted in significant decline in proliferation of glioma cells transfected with SALL4-siRNA (*P* < 0.05; Fig. 3a, b). However, this inhibition of proliferation was reversed by the PTEN inhibitor phen (bpv), here, this group we entitled SALL4-siRNA-bpv. Therefore, SALL4 can promote proliferation of glioma cells, and down-regulation of SALL4 could suppress cell growth.

### Down-regulation of SALL4 could induce cell cycle arrest at G1 phase

SALL4 could work as a promoter for tumor formation in various human tumors including glioma, but the mechanism was unclear. Cell cycle analysis was performed in U87 and U251 cells to better understand the cell cycle modulation. Outcomes revealed that G1 phase was increased and S phase was decreased (*P* < 0.05; Fig. 3c, d) in cells where SALL4 was blocked. And in cells with SALL4-siRNA-bpv, the repression effect was significantly relieved. Hence, inhibition of SALL4 expression in glioma by SALL4-siRNA arrested the cell cycle at G1 phase, and inhibited cell proliferation as seen by increased percentage of G1 phase cells and decreased S phase cells.

### Down-regulation of SALL4 could suppress the activation of PTEN/PI3K/AKT signaling pathway

**Fig. 4:**
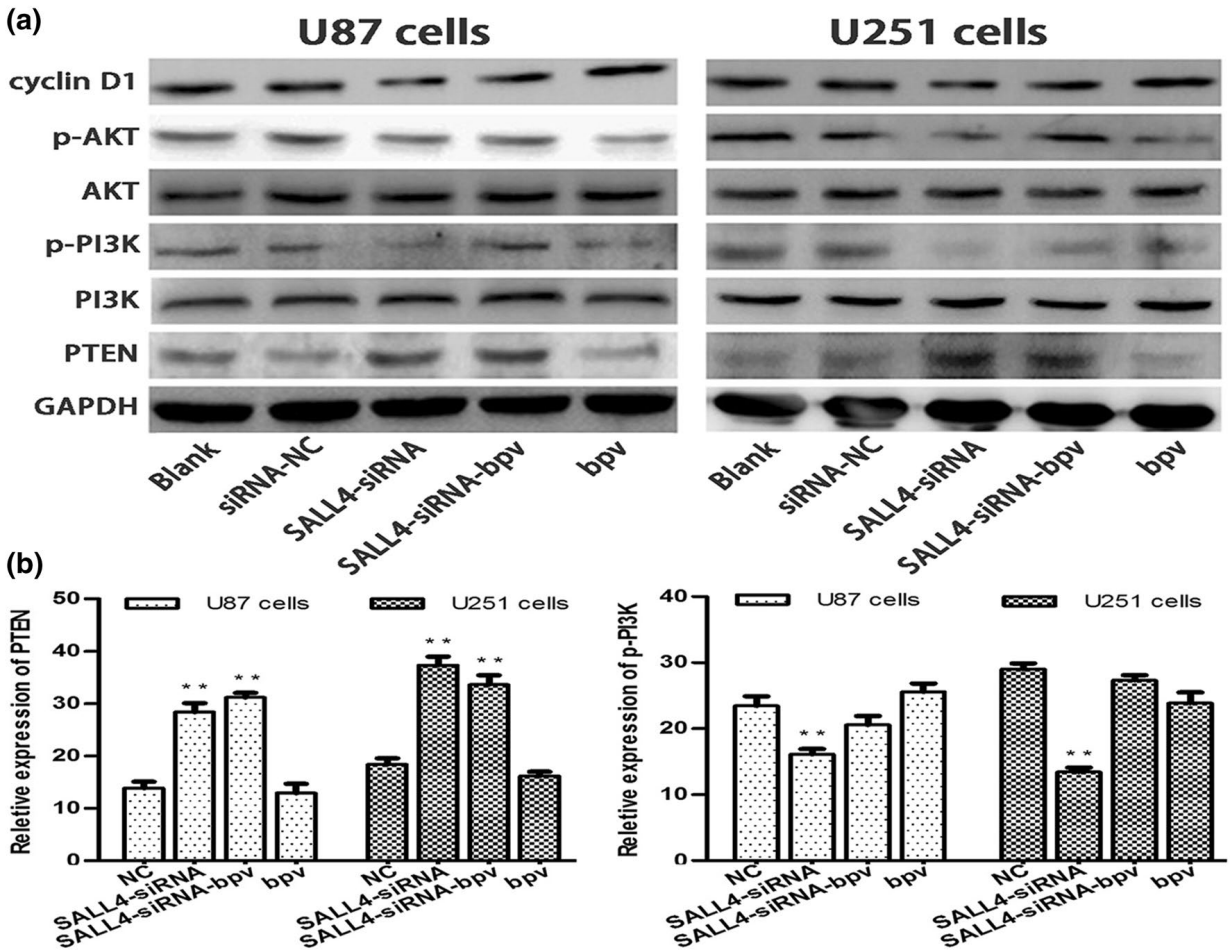
**a** The expression of SALL4, PTEN, PI3K, p-PI3K, AKT, p-AKT, cyclin D1 and GAPDH in different groups were detected by western blot analysis. The activity of PI3K/AKT signaling pathway was obviously decreased in cells transfected with SALL4-siRNA. **b** Immunofluorescence analysis data showed the expression of PTEN and p-PI3K after different treatment showed by bar graph

Down-regulation of SALL4 could increase the expression of PTEN. PTEN is an anti-tumor gene that depresses the PI3K/AKT signaling pathway in many cancers, but its role in glioma is unknown. In the present study, western blot results suggested that p-PI3K, p-AKT and cyclin D1 levels in cells transfected with SALL4-siRNA were significantly lower than in cells transfected with negative control, while the levels were moderate in SALL4-siRNA-bpv transfected cells (*P* < 0.05; Fig. 4a). Therefore, down-regulation of SALL4 expression suppressed activation of PTEN/PI3K/AKT pathway, which in turn impeded cell proliferation.

### PTEN and p-PI3K level was altered after blocking SALL4 in glioma cells

**Fig. 5:**
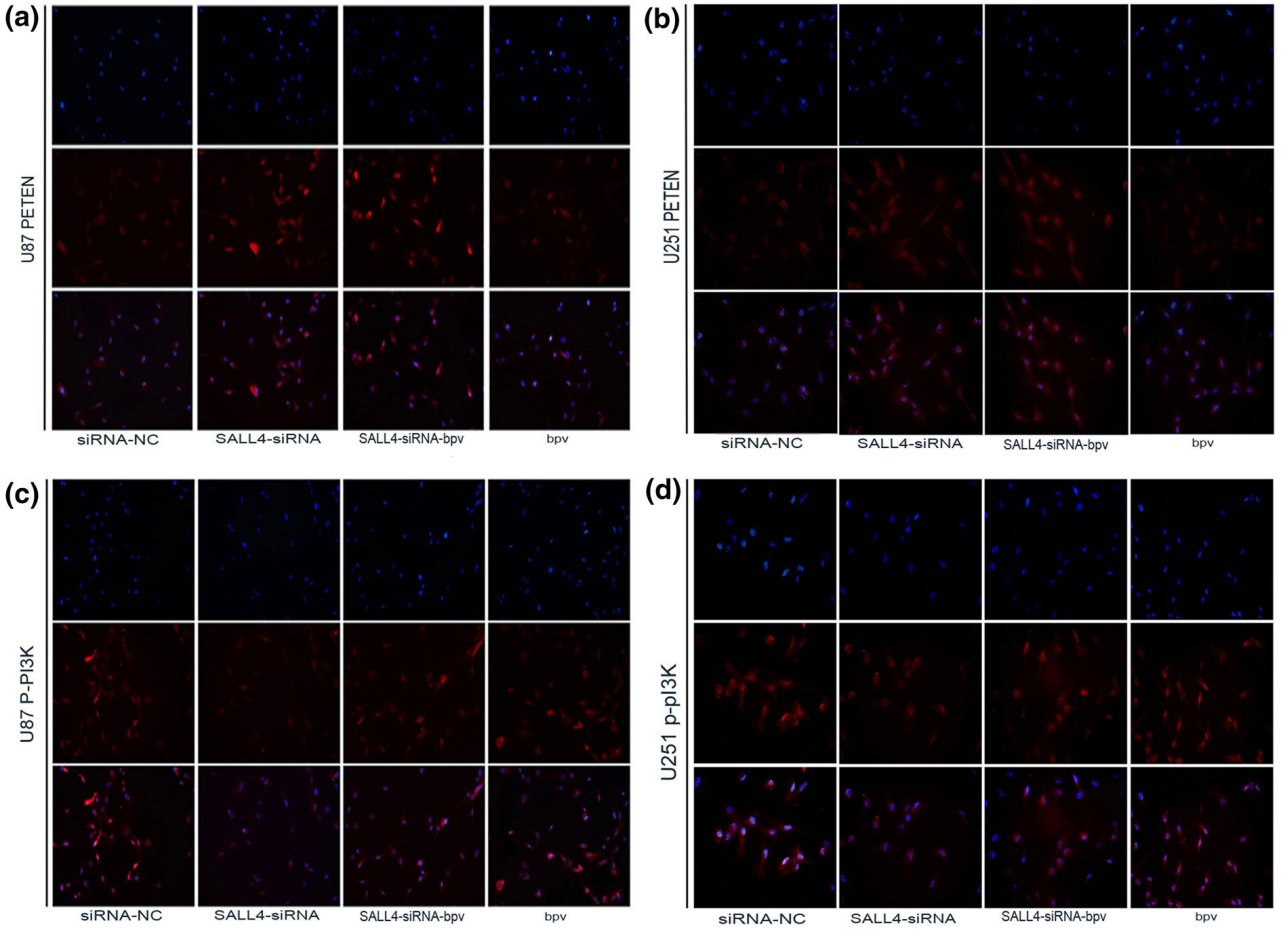
**a**–**d** Immunofluorescence analysis showed that the expression of PTEN was apparently increased and p-PI3K was inhibited in U87 and U251 cells after knocking down SALL4. However, PTEN inhibitor could reverse this effect

Immunohistochemistry showed that the expression of PTEN was increased, and p-PI3K was decreased in U87 and U251 cells after knocking down SALL4 as compared to the negative control group. When PTEN inhibitor phen (bpv) was added to the cells transfected with SALL4-siRNA, the protein level was similar to the negative control group (*P* < 0.05; Fig. 5a–d).

## Discussion

SALL4 is known to be abnormally expressed in multiple human tumors. In our study, abnormal expression of SALL4 was confirmed in glioma samples. Furthermore, blocking SALL4 increased PTEN expression and restrained the activation of PI3K/AKT pathway, thus suppressing cellular growth and proliferation of glioma.

SALL4 is expressed in the early stages of fetal development, and then diminishes as differentiation proceeds, with very low levels found in adults [22]. Up- or down-regulation of SALL4 is involved in cell proliferation, invasion, drug resistance, apoptosis, and other processes in some malignancies by targeting related genes [2, 23–26]. Owing to the strong proliferative function of SALL4 on cells and tissues, its correlation with various tumors was examined. SALL4 was aberrantly elevated in multiple carcinomas, such as leukemia, germ cell tumors, liver cancer and gastric cancer [2, 27, 28], acting as an oncogene and biomarker [23]. AJAY considered that SALL4 may be an extremely useful diagnostic marker in lung cancer. SALL4 played an important role in ESCs and human tumors. Abnormally high expression of SALL4 was closely related to tumor formation and prognosis in hematopoiesis and leukemogenesis, but SALL4 was also required for DNA damage response in ESCs, ensuring their stability during expansion [29]. SALL4 was a potent stimulator for the expansion of human hematopoietic stem/progenitor cells, esophageal squamous cell and gastric cancers [30, 31]. Over-expression of SALL4 enhanced gastric cancer cell proliferation and migration, whereas knocking down SALL4 reversed these effects [27]. Park found that SALL4 expression was significantly associated with a poor overall survival as compared to SALL4-negative HCCs [32], and implied poor prognosis in human hepatocellular and endometrial cancers. Down-regulation of SALL4 expression using small-hairpin RNA led to decreased in vitro myeloid colony-forming abilities and impaired in vivo engraftment in normal primary CD34+ cells [33]. SALL4 is a therapeutic target in intrahepatic cholangiocarcinoma (ICC) and endometrial cancer. However, its underlying mechanism during the development of ESCs and human tumors remains indistinct. Accumulating evidence reveals a crucial role for SALL4 in leukemogenesis due to its ability to promote proliferation and participation in Wnt/β-catenin pathways [34]. Wang believed that regulating NANOG, OCT4, and SOX2 may account for maintenance of pluripotent and self-renewal properties of ESCs. Hong W and Lauberth SM confirmed that by recruiting Mi-2/Nucleosome Remodeling and Deacetylase (NuRD) complex, SALL4 mediated transcription to impact cell growth [20].

PI3K/AKT signaling is a classical pathway involved in the regulation of tumorigenesis, hypoxia-induced VM formation, migration, invasion, and proliferation due to increase in the expression of phosphorylated EGFR (pEGFR), PI3K (p-PI3K), and AKT (p-AKT) [35–38]. Inhibitors of PI3K, for example, LY 294002 and PTEN, blocked the PI3K/AKT signaling and suppressed growth of diverse malignant tumors, including gliomas [14, 39]. Phen (bpv) is a known PTEN inhibitor that antagonizes PTEN function, and reverses the effect of PTEN as a cancer suppressor gene. Phosphorylated PI3K/AKT can promote cell growth and decrease apoptosis via increased Bcl-2/BAX ratio in human chondrosarcoma (CS) [40]. Cyclin D1 can be induced by growth factors through activation of various signaling pathways including PI3K/AKT, NF-kB [41–43], and p-AKT plays a crucial role in inducing cell proliferation by modulation of cyclin D1 in primary murine keratinocytes [43, 44]. In the present study, PTEN expression was up- regulation when SALL4 was reduced by siRNA-SALL4, as a result, the inhibitory action of PTEN on PI3K/AKT signaling was weaken, thus receded the cycling D1 level which arrested the cell cycle at G1 phase, regulating glioma proliferation. So, our study disclosed that SALL4 can inhibit PTEN and promote PI3K/AKT pathway in glioma, similar to leukemia [24].

In our text, SALL4 was found lower expression in some kinds of glioma samples than non-tumor brain tissues, a certain extent like the SALL4-positive immunoreactivity was 58% in total 102 intrahepatic cholangiocarcinoma cases [45]. Expression has been also reported SALL4 does not seem to be expressed in all trophoblastic tumors [46], individual genetic difference may be responsible for this phenomenon based on our relatively small sample, the more samples the more valuable outcome can be obtain.

## Conclusion

Our study showed significantly high expression of SALL4 mRNA in glioma specimens as compared to non-tumor samples using RT-PCR. Blocking SALL4 using SALL4-siRNA decreased proliferation of U87 and U251 cells, which was reversed by the addition of PTEN inhibitor phen (bpv). Furthermore, marked increase in PTEN mRNA and protein levels was seen in cells treated with siRNA-SALL4. These findings suggest that SALL4 can facilitate cell growth by suppressing PTEN expression in glioma cell lines. The PI3K/AKT activity was decreased in cells treated with pten (bpv) after transfecting with SALL4-siRNA, whereby p-PI3K and p-AKT protein levels were significantly increased as compared to cells transfected with SALL4-siRNA only. Therefore, blocking SALL4 could promote PTEN expression and restrain PI3K/AKT activity resulting in suppression of cell growth.
